# Physiological resilience of pink salmon to naturally occurring ocean acidification

**DOI:** 10.1093/conphys/coaa059

**Published:** 2020-07-31

**Authors:** Andrea Y Frommel, Justin Carless, Brian P V Hunt, Colin J Brauner

**Affiliations:** 1 Department of Zoology, University of British Columbia, Vancouver, BC, Canada; 2 Institute for the Oceans and Fisheries, University of British Columbia, Vancouver, BC, Canada; 3 Department of Earth, Ocean and Atmospheric Sciences, University of British Columbia,, Vancouver, BC, Canada; 4 Hakai Institute, Quadra Island, BC, Canada

**Keywords:** CO_2_, commercial fish, *Oncorhynchus*, Pacific, upwelling

## Abstract

Pacific salmon stocks are in decline with climate change named as a contributing factor. The North Pacific coast of British Columbia is characterized by strong temporal and spatial heterogeneity in ocean conditions with upwelling events elevating CO_2_ levels up to 10-fold those of pre-industrial global averages. Early life stages of pink salmon have been shown to be affected by these CO_2_ levels, and juveniles naturally migrate through regions of high CO_2_ during the energetically costly phase of smoltification. To investigate the physiological response of out-migrating wild juvenile pink salmon to these naturally occurring elevated CO_2_ levels, we captured fish in Georgia Strait, British Columbia and transported them to a marine lab (Hakai Institute, Quadra Island) where fish were exposed to one of three CO_2_ levels (850, 1500 and 2000 μatm CO_2_) for 2 weeks. At ½, 1 and 2 weeks of exposure, we measured their weight and length to calculate condition factor (Fulton’s *K*), as well as haematocrit and plasma [Cl^−^]. At each of these times, two additional stressors were imposed (hypoxia and temperature) to provide further insight into their physiological condition. Juvenile pink salmon were largely robust to elevated CO_2_ concentrations up to 2000 μatm CO_2_, with no mortality or change in condition factor over the 2-week exposure duration. After 1 week of exposure, temperature and hypoxia tolerance were significantly reduced in high CO_2_, an effect that did not persist to 2 weeks of exposure. Haematocrit was increased by 20% after 2 weeks in the CO_2_ treatments relative to the initial measurements, while plasma [Cl^−^] was not significantly different. Taken together, these data indicate that juvenile pink salmon are quite resilient to naturally occurring high CO_2_ levels during their ocean outmigration.

## Introduction

Climate change is causing the oceans to become warmer, more acidic and more hypoxic, as well as increasing the frequency, magnitude and duration of extreme events (Collins *et al.*, 2019, Gruber, 2011, Hegerl *et al.*, 2011, Jentsch *et al.*, 2007). While sea surface pH in the North Pacific is predicted to reach 7.7 by the end of the century, regional oceanographic conditions cause pH levels to drop below these levels today, such as in the California Current System and the Salish Sea (Feely *et al.*, 2008, Jiang *et al.*, 2019), and these extremes are expected to intensify with climate change (McNeil & Sasse, 2016, Pacella *et al.*, 2018). This acidification of the oceans has the potential to impact marine organisms from the individual to the ecosystem level (Fabry *et al.*, 2008).

Numerous studies have examined the effects of ocean acidification (OA) on marine organisms in an array of response variables, from gross measures of survival and growth rates, development and behaviour, to calcification, metabolism and gene expression. Responses to high CO_2_ in fishes, however, vary widely from strong negative effects, to no effects and even positive effects (Cattano *et al.*, 2018). One of the main reasons for the diversity of responses is likely local variability of ocean climate on small temporal and spatial scales (Helmuth *et al.*, 2014, Waldbusser *et al.*, 2014), such that pre-exposure to naturally CO_2_-enriched habitats may provide a buffer to future acidification through local adaptation and phenotypic plasticity (Baumann, 2019). This has been demonstrated in the response to CO_2_ by marine invertebrates inhabiting areas of graded upwelling regimes of the coast of Chile (Vargas *et al.*, 2017). Studies on fish have shown differences in CO_2_ vulnerability in two populations of Atlantic cod (*Gadus morhua*): larvae of the Norwegian coastal population that develop in stable, ambient CO_2_ conditions were vulnerable to near-future OA (Frommel *et al.*, 2012), whereas larvae of the Baltic population that develop in naturally high CO_2_ water were resilient to levels up to 4000 μatm CO_2_ (Frommel *et al.*, 2013). Even within a population, seasonal changes in ocean conditions can affect the vulnerability of individuals to experimental OA (Murray *et al.*, 2014). On the other hand, some studies have found species living in naturally enriched CO_2_ areas to be robust to current levels of CO_2_, but highly vulnerable to higher levels (Thomsen *et al.*, 2010). Whether organisms living in areas of naturally high CO_2_ will be more resilient to future climate change or whether the added acidification represents a tipping point is still unclear and likely to be ecosystem, species or even life-stage specific.

The eastern North Pacific is a hotspot for OA, and CO_2_ levels >1000 μatm are seasonally measured on the continental shelf in association with upwelling, in inland coastal areas, and estuarine habitats (Evans *et al.*, 2019, Feely *et al.*, 2010). The Salish Sea, at the southern end of British Columbia, is a semi-enclosed estuary due to narrow connections with the open Pacific in the north (Queen Charlotte Strait) and south (Juan de Fuca Strait) and a large freshwater discharge, mainly from the Fraser River. Water circulation is driven by tides and winds and is strongly affected by the bathymetry, as well as seasonal cycles with interannual variability in spring and summer pH and CO_2_ levels from river discharge (Ianson *et al.*, 2016, Moore-Maley *et al.*, 2016). Modelling simulations have shown that corrosive (Ω_aragonite_ < 1) conditions in the Northern Salish Sea are driven by anthropogenic CO_2_ emissions, were likely absent before 1900 and will likely be exacerbated below biological thresholds for vulnerable species during most of the year (Evans et al., 2019).

With the Fraser River watershed, British Columbia has one of the world’s largest salmon-producing river systems. However, since the 1990s, stocks of Pacific salmon have been in decline, with climate change named as one of the main contributing factors (Cohen, 2012, Riddell *et al.*, 2013). Salmon are particularly vulnerable during their early life history, and high mortalities during this time may be responsible for interannual variability and long-term declines in salmon recruitment (Friedland *et al.*, 2000, Mueter *et al.*, 2002). Anadromous salmon migrate out of freshwater into seawater as juveniles, a process that requires complete remodelling of their physiology for osmoregulation, acid-base status, respiration, circulation and metabolism, termed smoltification (Hoar, 1988), to prepare them for life in the ocean (Hoar, 1988, Høgåsen, 1998). High mortality at this life stage has been linked to a combination of suboptimal ocean conditions and poor food availability during an energetically costly life stage (Beamish *et al.*, 2001 , McKinnell *et al.*, 2014, Mueter *et al.*, 2005).

Pink salmon (*Oncorhynchus gorbuscha*) migrate to the ocean at the earliest developmental stage and the smallest size of all Pacific salmon. Their small size creates challenges for osmotic and ion regulation, as they have a high surface area to volume ratio. Additionally, they migrate into seawater prior to completing smoltification and their gill Na^+^K^+^-ATPase (NKA) activity continues to increase for weeks following seawater entry (Gallagher *et al.*, 2012, Grant *et al.*, 2009). Gill ion- and osmoregulation may be further challenged by elevated environmental CO_2_ conditions associated with additional acid-base regulatory challenges that may impact subsequent condition and performance. Juvenile pink salmon exhibit up to a 30% reduction in maximum oxygen consumption rate after 2 weeks in high CO_2_ seawater (1600 μatm CO_2_) independent of previous freshwater CO_2_ exposure (10 weeks in either 450 or 2000 μatm CO_2_), indicating no carry-over effects from the freshwater to seawater phase (Ou *et al*., 2015). From this study, it was hypothesized that pink salmon may be particularly vulnerable to OA based on their unique life-history strategy.

**Figure 1:**
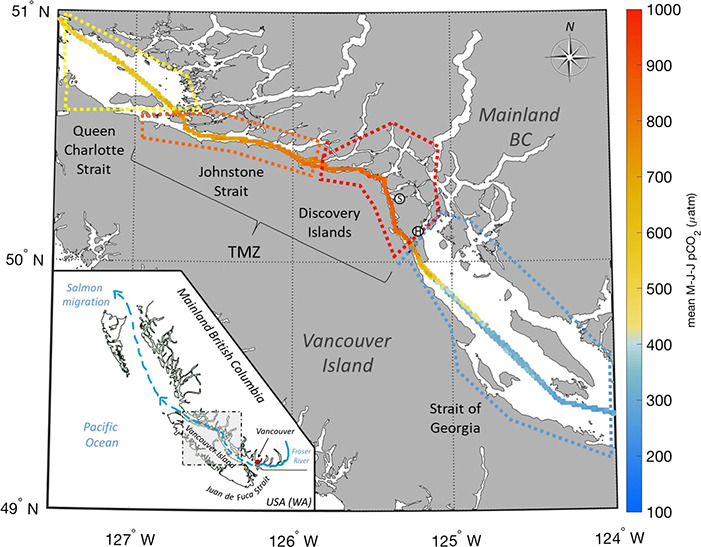
Mean sea surface *p*CO_2_ data in the Salish Sea during peak salmon migration May–July from ferry observations, gridded by 1 km^2^ (23 transits over 2 years), from the southern Strait of Georgia through the TMZ (Discovery Islands + Johnstone Strait) to the Queen Charlotte Strait. Data from Wiley Evans, Hakai Institute. Location of salmon capture in Granite Bay indicated by (S); Hakai Institute’s Quadra Island Field Station indicated by (H). Map inset: juvenile salmon migration route from the Fraser River, through the Salish Sea, to the Pacific Ocean.

This is especially relevant for Fraser River salmon, as they experience high CO_2_ conditions, high temperature and low O_2_ during their seaward migration. After entering the ocean in early May, a key migration route leads Pacific salmon from the northern Strait of Georgia through Johnstone Strait and Queen Charlotte Strait before entering the Gulf of Alaska (Welch *et al.*, 2011), exposing them to highly varied hydrographical conditions along the way (Fig. 1). The Strait of Georgia is a complex estuarine habitat, with the southern region strongly influenced by the Fraser River plume. This freshwater input lowers the total alkalinity of the water, weakening the buffering capacity for CO_2_ acidification (Evans *et al*., 2019). In the central and northern Strait of Georgia, there is a distinct seasonal cycling in *p*CO_2_ with warm, stratified and productive waters in summer with CO_2_ levels around 300 μatm and highly mixed in winter with *p*CO_2_ levels around 700 μatm (Evans *et al*., 2019). However, this system is also characterized by periodic upwelling events due to strong northwesterly winds, when *p*CO_2_ levels above 1000 μatm can be measured at the sea surface in winter and spring during peak salmon migration (Tortell *et al.*, 2012). After leaving the Strait of Georgia, the salmon pass a sill into the Discovery Islands, where the water is persistently deeply mixed. Cold, dense and CO_2_-rich deep water is mixed through the surface and this region has been termed the tidal mixing zone (TMZ). The TMZ is particularly challenging both in terms of oceanographic and ecological conditions where persistent winds and tidal mixing elevate the *p*CO_2_ of the entire water column (Evans *et al.*, 2019), as well as create inadequate food supply (Stormer *et al.*, 2016, Thomson *et al.*, 2012). Field data shows that juvenile salmon have low to no foraging success in the TMZ (Discovery Islands and Johnstone Strait), due to unfavourably small prey (James *et al.*, 2020) leading to mean gut fullness index (GFI; to % body weight) of 0.41% in pink salmon across the TMZ with up to 40% empty guts (Fladmark, unpublished). Based on tagging studies with sockeye salmon (Rechisky *et al.*, 2017) and swim speeds of juvenile pink salmon (Nendick *et al.*, 2011), travel time from the entrance to the TMZ to the exit of the Johnstone Strait is estimated to be a minimum of 2 weeks for juvenile pink salmon, exposing them to these unfavourable conditions for the duration of our experiment.

To investigate the effects of ocean acidification on juvenile salmon physiology during their migration through naturally CO_2_ enriched waters, we caught wild juvenile pink salmon at the entrance to the TMZ and exposed them to three levels of CO_2_ (850, 1500 and 2000 μatm CO_2_) for 2 weeks at the Hakai Institute’s Quadra Island Field Station experimental facility. Fish were sampled at regular intervals to determine condition, haematocrit and plasma [Cl^−^] to gain insight into osmoregulatory and acid-base status. At each sampling time, fish were subjected to additional stressors associated with climate change—namely, thermal and hypoxia challenge, to provide a more comprehensive picture of the overall physiological condition of the fish exposed to these CO_2_ treatments.

**Table 1:** Mean +/− SD carbonate chemistry parameters: temperature, salinity and pH_Total_ from bi-daily measurements with a hand-held multimeter (VWR) TCO_2_ and *p*CO_2_ measured at distinct sampling intervals with the BoL; TA calculated with CO2SYS

**Treatment**	**Temperature (°C)**	**Salinity**	**pH**_**Total**_	***p*CO**_**2**_ **(μatm)**	**TCO**_**2**_ **(μmol/kg)**	**TA (μmol/kg)**
Control	13.07 ± 0.61	30.70 ± 0.11	7.72 ± 0.02	856.70 ± 73.85	1996.16 ± 10.39	2050.73 ± 9.77
Medium	13.14 ± 0.54	30.70 ± 0.06	7.53 ± 0.02	1520.47 ± 133.22	2080.29 ± 17.33	2074.53 ± 7.41
High	12.94 ± 0.50	30.72 ± 0.15	7.41 ± 0.03	2042.33 ± 235.99	2106.13 ± 24.48	2064.94 ± 11.06

## Methods

### Fish capture and husbandry

Juvenile pink salmon (500 fish, 67–87 mm fork length, 2.75–4.3 g body weight) were caught with a juvenile fish seine in Granite Bay (50°15′27.3″N 125°20′50.1″W) off the west coast of Quadra Island in the TMZ of the Discovery Passage between the Strait of Georgia and the Johnstone Strait. Fish were held in aerated water in a large holding tank and immediately transported back to the Marna Laboratory at the Hakai Institute’s Quadra Island Field Station. Here, 40 fish were placed in each of 9 large (90 L) holding tanks, each provided with flow-through (300 ml min^−1^) filtered and UV sterilized seawater taken directly from the bay (at 20 m depth) in front of the lab. Three of the 90 L large holding tanks were held within each of three large water baths (5000 L) maintained at 13°C using industrial chillers. The salinity remained between 29 and 30 for the duration of the experiment in all 90 L holding tanks. Before the start of the experiment, fish were allowed to acclimate to the 90 L holding tanks for 24 h after which a baseline sample was taken from 29 fish for wet weight, length, blood, haematocrit and plasma (see below). After 24 h, each of the 9–90 L tanks was randomly assigned one of three CO_2_ treatments: control CO_2_ (compressed air only), medium CO_2_ (1500 μatm CO_2_) and high CO_2_ (2000 μatm CO_2_), each replicated three times. Target CO_2_ levels were achieved using mass-flow controllers bubbling premixed air/CO_2_ at 0.8 L min^−1^ into each tank. The fish were held for 2 weeks under these conditions with measurements of thermal and hypoxia tolerance conducted at ½, 1 and 2 weeks of CO_2_ exposure (see below). The fish were not fed during this 2-week exposure to mimic poor natural ocean feeding conditions (Fassbender, unpublished), as well as to prevent confounding effects of potential CO_2_-induced differences in feeding success. No mortalities (outside of scheduled sampling-induced mortalities) occurred during the course of the experiment. Water was measured twice a day (morning and evening) for temperature, salinity, pH and O_2_ using a handheld multimeter (VWR H30PCD), and oxygen saturation remained above 92% in all tanks (mean 96% ± 4.3% O_2_). Ammonia levels were measured three times a week with a water test kit and remained below 0.1 mg/L in all tanks. Water samples were taken at the beginning, middle and end of the experiment and analyzed for carbonate chemistry using a Burke-o-Lator *p*CO_2_/TCO_2_ analyzer (BoL, Dakunalytics, LLC) following the approach described in Evans et al., 2019. Briefly, *in situ p*CO_2_ and pH_Total_ were calculated from directly measured *p*CO_2_, TCO_2_, temperature and salinity using CO2SYS with the carbonic acid dissociation constants (K1 and K2) from Luecker *et al*., 2000; the KHSO4 dissociation constant from Dickson *et al*., 1990; the boron/chlorinity ratio from Uppstrom, 1974; and the aragonite solubility constant from Mucci, 1983. Control water was higher than the global average of 400 μatm due to the water being taken out of the Bay in the TMZ that is persistently CO_2_-enriched. For a summary of the carbonate parameters, see Table 1.

### Condition (Fulton’s *K*)

Wet weight and fork length were measured in all fish sampled. The Fulton’s condition factor (*K*) was determined by dividing the weight by the cubed length.

### Haematocrit and plasma [Cl^−^]

A subset of five fish per tank were randomly sampled at 1, 4, 8 and 14 days of CO_2_ exposure (referred to as baseline, ½, 1 and 2 weeks from this point forward) and euthanized with an overdose of buffered MS222 (500 mg/L). The caudal peduncle was severed, and blood was collected from the caudal vein directly into 75-mm heparinized haematocrit tubes in triplicate per fish. Tubes were spun down with a haematocrit centrifuge and callipers were used to measure the height of packed red blood cells (RBCs) which was divided by the total height of RBCs and plasma combined to yield haematocrit. The haematocrit tube was scored at the interface of the RBCs and plasma, and plasma was expelled into 0.5 ml of microcaps and frozen in liquid nitrogen for later measurement of plasma [Cl^−^]. Immediately prior to analysis, plasma samples were thawed and centrifuged. Plasma [Cl^−^] was measured in duplicate (provided there was sufficient plasma) with coulometric titration (ChloroChek® Chloridometer®).

### Acute upper thermal tolerance

Acute upper thermal tolerance was measured by the critical thermal maximum (CTmax) using the standard methodology for fish (Beitinger *et al.*, 2000). On Days 1, 3, 7 and 13 of CO_2_ exposure (referred to as baseline, ½, 1 and 2 weeks from this point forward), a subsample of five fish per tank (sampled in random order) were transferred to 3 × 20 L plastic aquaria filled with water from their respective CO_2_ treatment and covered with black foil to reduce stress. An air-stone supplied from the respective mass-flow controllers was used to keep *p*CO_2_ relatively constant as the temperature was increased, as well as to help mix the water to create a homogeneous water temperature throughout the tank. After a 1-h acclimation period, the previously inserted heat stick was turned on, and the water was heated at a constant rate of 0.3°C min^−1^ until loss of equilibrium (LOE) (Jung, 2018). The temperature was measured using high-accuracy digital thermometers (Hanna Checktemp1) with values manually recorded every 5 min to track the rate of heating. The end-point LOE was determined when the individual fish was not able to right itself when gently prodded with forceps, at which point the temperature (CTmax) and time were noted, and the fish was quickly removed and euthanized with an overdose of buffered MS222 (500 mg/L).

**Figure 2:**
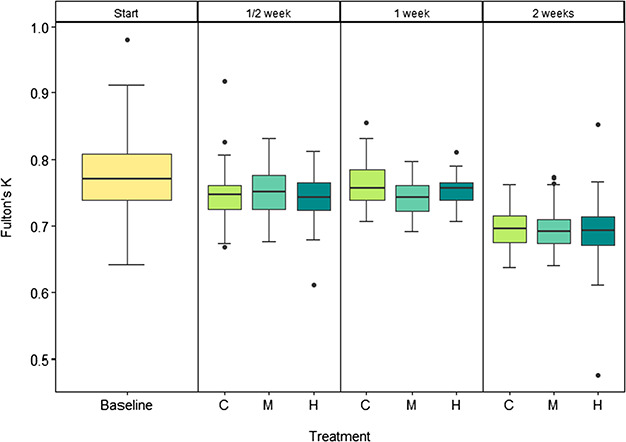
Box-whisker plots of Fulton’s *K* index of condition at the beginning (baseline, yellow) and after ½, 1 and 2 weeks in control (C: 850 μatm CO_2_, light green), medium (M: 1500 μatm CO_2_, aquamarine) and high (H: 2000 μatm CO_2_, dark cyan) CO_2_ treatments. *n* = 15.

### Hypoxia tolerance

Hypoxia tolerance was determined from the incipient lethal oxygen saturation (ILOS) (Claireaux and Chabot 2016). A subset of five fish per CO_2_ treatment tank were sampled on Days 1, 3, 7 and 13 of CO_2_ exposure (referred to as baseline, ½, 1 and 2 weeks from this point forward) and transferred to a 20 L covered plastic aquaria with water from the respective CO_2_ treatment tank. After a 1-h acclimation, medical grade nitrogen was bubbled into the tank via an air-stone to reduce the water oxygen saturation by ~0.3% min^−1^ (Jung, 2018). Oxygen levels were measured with a calibrated portable O_2_ meter (YSI) and recorded every 5 min to monitor the rate of O_2_ decrease. The incipient lethal oxygen saturation (ILOS) was determined as percent oxygen saturated air (100% O2 refers to air saturation) at which the fish lost equilibrium and was not able to right itself in response to gentle prodding with forceps. At this point, % O_2_ and the time were noted, and the fish was quickly removed and euthanized with an overdose of buffered MS222 (500 mg/L).

### Statistics

All statistics were performed in R (R Core Team, 2018), and graphics were created using ggplot. *P*-values were generated using MANOVAs and Pillai’s trace test to account for type 1 error with small sample sizes. *Post hoc* comparisons were run on separate sampling days. Beginning (baseline) and final values (after 2-week CO_2_ exposure) were compared for each treatment with an effect size analysis, using the natural logarithm of the response ratio (LnRR) and calculating 95% confidence intervals (Hedges & Olkin, 1985).

### Animal care

This study was carried out under strict accordance with the animal care protocol approved by the University of British Columbia Animal Care Committee (certificate # A15-0266). Fish were collected under the Fisheries and Oceans permit number XR 63 2019.

## Results

### Mortality

No mortality occurred in any of the treatments, and thus, 100% of the fish survived all of the CO_2_ treatments for the entire 2-week duration of the experiment.

### Condition

Fulton’s index of condition (Fulton’s *K*) was significantly affected by both treatment (*F*_3_ = 15.093; *P* < 0.001) and date (*F*_1_ = 116.836; *P* < 0.001). A *post hoc* test revealed that this was due to the decrease in condition relative to the baseline (Tukey HSD, *p*_adj_ < 0.001 for all treatments). After 2 weeks, *K* was reduced by 10% in all treatments relative to the baseline (Fig. 2). Excluding the baseline from the ANOVA showed no significant difference in treatment over the course of the experiment (*F*_2_ = 1.58; *P* = 0.207). Table 2 summarizes the wet weights for fish throughout the experiment.

**Table 2:** Mean ± SD of wet weights for initial sample (start) and after ½, 1 and 2 weeks of experiment for three different CO_2_ treatments

Treatment	Start	½ week	1 week	2 weeks
Baseline	3.49 ± 0.43			
Control		3.57 ± 0.44	3.54 ± 0.56	3.32 ± 0.34
Medium		3.89 ± 0.59	3.60 ± 0.46	3.12 ± 0.69
High		3.42 ± 0.58	3.72 ± 0.53	3.34 ± 0.45

### Upper thermal maximum (CTmax)

**Figure 3:**
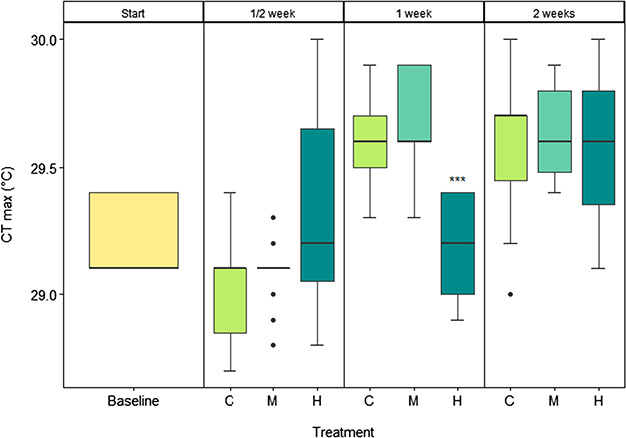
Upper thermal maximum (CTmax) of juvenile pink salmon as measured by the temperature at which the fish lost equilibrium (LOE) at the start of the experiment (baseline, yellow) and after a ½, 1 and 2 weeks of exposure to control (C, light green), medium (M, aquamarine) and high (H, dark cyan) CO_2_ treatments. *n* = 15. Significance code: 0 ‘^***^’ 0.001.

Overall, there was a significant effect of treatment (*F*_3_ = 4.758; *P* = 0.00388), days of exposure (*F*_1_ = 47.049; *P* > 0.001) and the interaction (*F* = 12.921; *P* > 0.001) on CTmax (Fig. 3). There was a significant difference between treatments only after 1 week of exposure, with fish in the high CO_2_ treatment losing equilibrium at a significantly lower temperature than the control and medium CO_2_ treatments (*F*_2_ = 14.21, *P* > 0.001), indicating a lower thermal tolerance. The weight of the fish was not significant (*F*_1_ = 0.536, *P* = 0.466) when included in the model.

### Hypoxia tolerance (ILOS)

Overall ILOS increased by an average of 25% from the start to 2 weeks, with a significant difference in treatment (*F*_3_ = 4.758; *P* = 0.00388), day (*F*_1_ = 47.049; *P* < 0.001) and the interaction of both (*F*_2_ = 12.921; *P* < 0.001). A *post hoc* test showed that, after 1 week of exposure, ILOS in the medium and high CO_2_ treatments were significantly higher relative to the control (*F*_2_ = 78.75; *P* > 0.001) with the medium CO_2_ treatment exhibiting a 45% increase in ILOS and the high CO_2_ treatment a 25% increase. After ½ and 2 weeks, the treatment levels were not significantly different to the control group.

### Haematocrit

Haematocrit was not different between the treatments after ½ and 1 week, but mean haematocrit significantly increased by 16% in the high CO_2_ treatment relative to the control CO_2_ after 2 weeks of exposure (*F*_2_ = 4.38; *P* = 0.0187).

### Plasma [Cl^−^]

There was no significant difference in plasma [Cl^−^] between the treatments (*F*_3_ = 1.636; *P* = 0.186) or day (*F*_1_ = 0.444; *P* = 0.507).

### Overall effect of the 2-week exposure to elevated CO_2_ levels

An effect size analysis of the different CO_2_ treatments relative to the baseline after 2 weeks of exposure shows the overall effect in the different factors examined (Fig. 7). After 2 weeks, the condition of the juvenile salmon was reduced by a mean of 10% in all treatments relative to the baseline. CTmax was increased by a mean of 1% in all treatments relative to the baseline, while ILOS was significantly increased by a mean 30, 23 and 18% in the control, medium and high treatment, respectively. Haematocrit was significantly higher in both the medium and high treatments relative to the baseline (mean 17 and 22%, respectively), while the control was not significantly different. Plasma [Cl^−^] levels were not significantly different between the baseline and any treatment after 2 weeks but had a large individual variation.

## Discussion

Despite our predictions that juvenile pink salmon would be vulnerable to high levels of CO_2_ during their energetically costly phase of seaward migration, our data indicate that juvenile pink salmon appear to be quite robust to the levels of CO_2_ exposure used in this study. Although the fish were exposed to CO_2_ concentrations up to 2000 μatm for 2 weeks in this experiment and not fed, there was no mortality. Their condition factor (Fulton’s *K*) decreased an average of 10% by the end of the experiment; however, this was not affected by CO_2_ treatment (Figure 7) and likely the result of food deprivation. Significant reductions in tolerance to additional acute stressors of high temperature or low oxygen were only seen after 1 week of exposure, but not after ½ or 2 weeks. Haematocrit was slightly increased in the high CO_2_ treatment level relative to the control after 2 weeks of exposure, possibly indicating an enhanced capacity for oxygen transport that may be associated with stress. Elevated haematocrit due to an increase in red blood cells aids oxygen delivery to the tissues. Therefore, higher oxygen delivery to the brain and nervous system may have postponed loss of equilibrium in the ILOS and CTmax trials after 2 weeks in the medium and high CO_2_ treatments, leading no significant differences between control and high CO_2_ treatments. Plasma [Cl^−^], however, remained unchanged over the experimental period indicating a stable osmoregulatory status with CO_2_ exposure. Together, these findings indicate that levels of CO_2_ up to 2000 μatm for 2 weeks at this life stage of pink salmon are not associated with physiological impairment as measured in this study and have only a small effect on simultaneous acute stressors.

While many studies find no effects of OA on fish (Cattano *et al*., 2018), negative effects of elevated CO_2_ have been demonstrated for salmon in both fresh- and saltwater for growth and survival, behaviour and olfaction in larval and juvenile stages (Ou *et al*., 2015, Williams *et al.*, 2019). Ou et al. (2015) showed a decrease in maximal metabolic rate and aerobic scope in smolting pink salmon exposed to 2000 μatm CO_2_, irrespective of previous freshwater CO_2_ exposure. Since aerobic scope and tolerance to low oxygen and high temperature have been correlated in salmon, with a proposed functional link through oxygen supply (Zhang *et al.*, 2018), we predicted that hypoxia and thermal tolerance may be reduced in salmon at higher CO_2_ concentrations.

**Figure 4:**
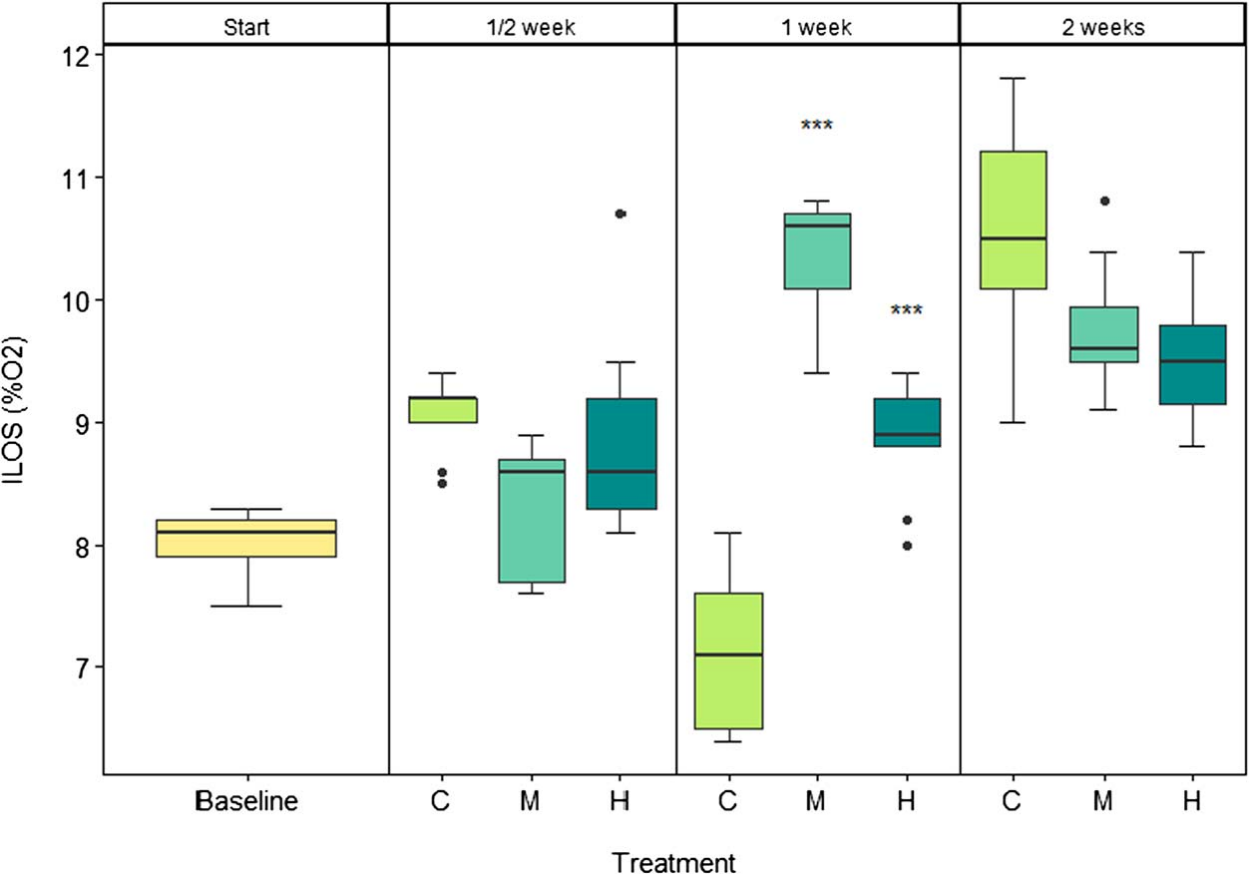
Hypoxia tolerance (ILOS) of juvenile pink salmon as measured by the % oxygen saturation (100% O_2_ refers to air saturation) at which the fish loses equilibrium (LOE) at the start (baseline, yellow) of the experiment and after a ½, 1 and 2 weeks of exposure to control (C, light green), medium (M, aquamarine) and high (H, dark cyan) CO_2_ treatments. *n* = 15. Significance code: 0 ‘^***^’ 0.001.

CTmax was only significantly reduced in the high CO_2_ treatment after 1 week of exposure (Figure 3). However, this difference appears to be largely driven by an increased thermal tolerance in the control and medium CO_2_ treatment compared to the start and ½ week of exposure and thus may signal a lack of adjustment in the high CO_2_ treatment compared to the control and medium treatment. Interestingly, this significant difference in thermal tolerance following 1 week of exposure to high CO_2_ was also associated with a reduction of hypoxia tolerance. After 1 week of CO_2_ exposure, the medium and high CO_2_ levels showed a 25 and 45% increase in ILOS, respectively (Figure 4). However, this significant difference was largely driven by a lower ILOS in the control treatment, relative to the medium and high CO_2_ exposure. If thermal tolerance and hypoxia tolerance are functionally related, a reduction in thermal performance at high temperatures may be the result of oxygen limitation (Anttila *et al.*, 2013) as is the case for hypoxia tolerance. Thus, while it is interesting that there are significant reductions in both thermal and hypoxia tolerance at 1 week of CO_2_ exposure, the basis is unclear and further studies should be conducted to clarify this.

**Figure 5:**
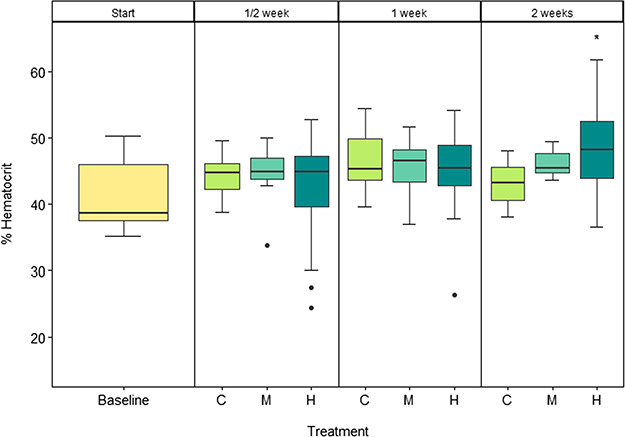
Haematocrit in juvenile pink salmon at the start (baseline, yellow) and after 1/2, 1 and 2 weeks of exposure to control (C, light green), medium (M, aquamarine) and high (H, dark cyan) CO_2_ treatments. *n* = 15. Significance code: 0.01 ‘^*^’ 0.05.

**Figure 6:**
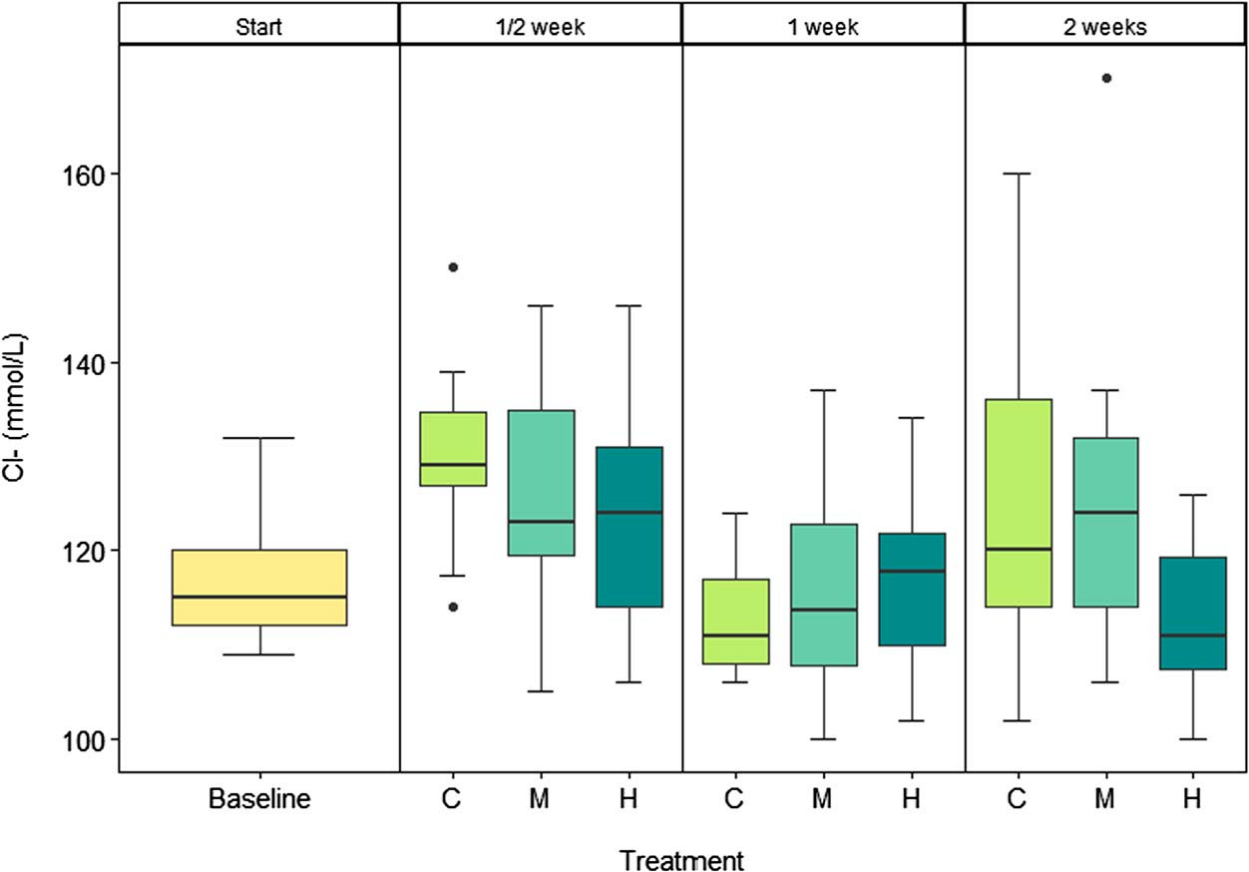
Plasma [Cl^−^] in juvenile salmon at the start (baseline, yellow) and after 1/2, 1 and 2 weeks of exposure to control (C, light green), medium (M, aquamarine) and high (H, dark cyan) CO_2_ treatments. *n* = 11–15.

To compensate for a CO_2_-induced acidosis, fish generally elevate plasma [HCO_3_^−^] in exchange for [Cl^−^], which is associated with net proton excretion (Brauner *et al.*, 2019). The increase in plasma [HCO_3_^−^] during CO_2_ exposure is matched with an equimolar reduction in plasma [Cl^−^]. In our study, no differences in plasma [Cl^−^] were detected between CO_2_ treatments at any sampling day (Figure 6). As plasma [Cl^−^] was very variable within each treatment and the expected changes in [Cl^−^] at 2000 μatm *p*CO_2_ relative to our control treatment would be <2 mmol/L (Brauner *et al.*, 2019, Heuer *et al.*, 2016), which may be below our ability to detect this change. Plasma [Cl^−^] could also be affected by osmoregulatory status; however, given that no significant changes were observed, it does not appear that this CO_2_ level resulted in any negative osmoregulatory effects.

**Figure 7:**
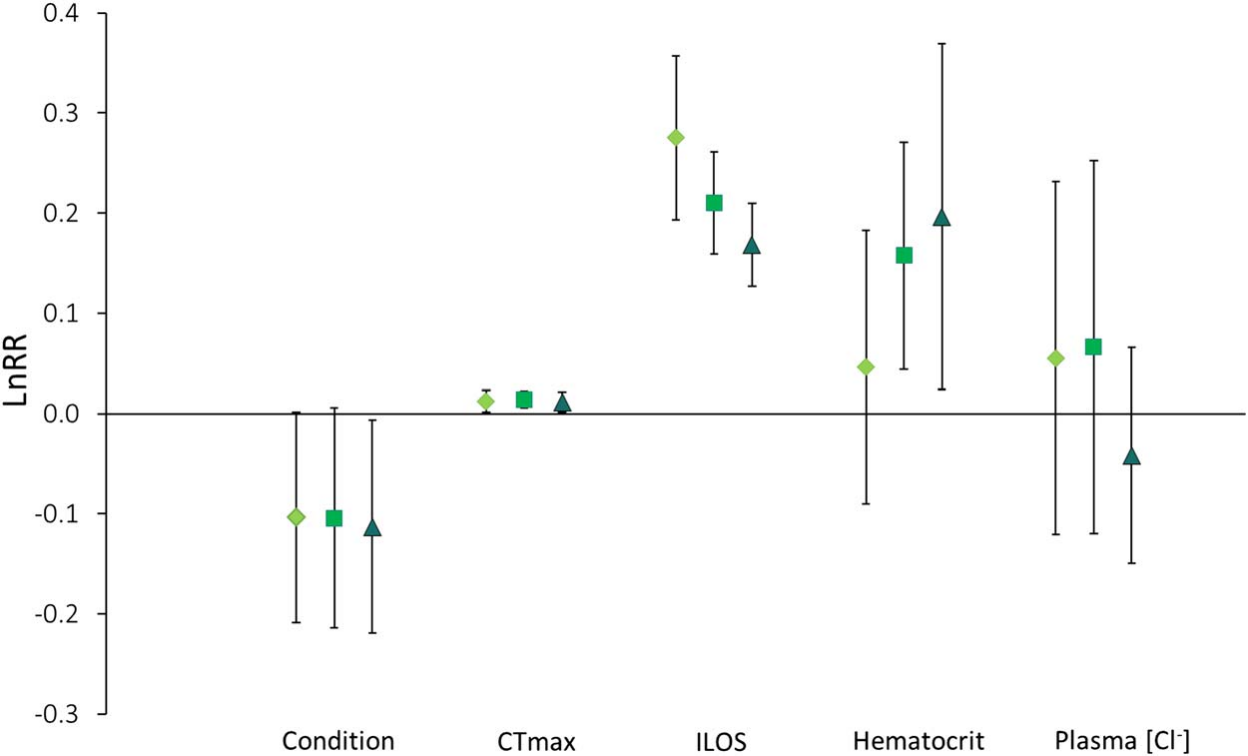
Effect size analysis using the natural logarithm of the response ratio (LnRR) of the three CO_2_ levels after 2 weeks of exposure relative to the baseline: control (diamond, light green), medium (square, aquamarine) and high (triangle, dark cyan) treatment for condition, thermal tolerance (CTmax), hypoxia tolerance (ILOS), haematocrit and plasma [Cl^−^]. Mean values with 95% confidence intervals. Effect is significant if the error bar does not cross zero.

Following a 2-week exposure to high CO_2_, there was a statistically significant increase in haematocrit (Figure 5), which may have been due to either an elevation in red blood cell numbers or associated with adrenergic stimulation of red blood cells (Nikinmaa, 1990). The elevated haematocrit could have been the compensatory mechanism improving ILOS and CTmax in the CO_2_ treatments after 2 weeks of exposure through enhancing O_2_ unloading (Brauner & Wang, 1997, Rummer *et al.*, 2013). While there was a decrease in hypoxia tolerance by an average of 25% after 2 weeks, compared with the baseline, there was no difference between the treatment levels (Figure 7). This could indicate accumulation of physiological effects of stress in the fish due to confinement, as well as a starvation effect. While loss of equilibrium during CTmax trials seem to be controlled by neural failure (Jutfelt et al., 2019), the end-point of ILOS generally occurs when adenosine triphosphate (ATP) levels in the brain are depleted (Speers-Roesch *et al.*, 2013). As ATP production is highly dependent on glycogen stores, a fish that has not fed for 2 weeks will likely have lower glycogen stores, leading to LOE sooner at higher oxygen concentrations. This was reflected in the condition factor that showed an average 10% decrease in all treatments after 2 weeks relative to the baseline (Figure 7). Therefore, the reduction in glycogen stores after 2 weeks without food may be masking more subtle effects of CO_2_ exposure. Future studies should include fed fish during CO_2_ exposure to investigate the effects of food deprivation and water chemistry on tolerance assays in juvenile salmon.

However, studies looking at the gut contents have shown that like juvenile sockeye (James, 2019), pink and chum feed poorly during their migration through the TMZ, with a large proportion (up to 40%) having empty stomachs throughout most of this region (Fladmark, unpublished data). This has been linked to depressed growth of fish sampled in the Johnstone and Queen Charlotte Strait, indicating a lower condition compared to salmon caught in the Strait of Georgia (Ferriss *et al.*, 2014, Journey *et al.*, 2018). Suboptimal ocean conditions, an altered Redfield ratio, and low plankton abundance and diversity may all be contributing factors to low salmon foraging success in this area (Mackas *et al.*, 2007, Preikshot *et al.*, 2013, Schweigert *et al.*, 2013). After exiting the TMZ, feeding commences and growth and condition improve (Ferriss *et al*., 2014). The travel time of juvenile salmon through this region is estimated to be around 2 weeks (the duration of our study), based on telemetry data available for sockeye salmon (Rechisky et al. 2017). Therefore, 2 weeks of elevated CO_2_ conditions and food deprivation are naturally encountered by juvenile salmon during this section of their migration and may have primed them to compensate these conditions for the duration they experience them in the wild.

Salmonids are well adapted to periods of fasting and are able to fully remobilize metabolic reserves when food becomes available again (Navarro & Gutiérrez 1995). From several studies, we know juvenile salmonids have a high resistance to starvation, with survival up to 13 weeks of starvation in juvenile chum (Akiyama & Nose, 1980) and actively swimming rainbow trout, (Simpkins *et al.* 2003), with hormonal and weight responses generally setting in after 2-weeks of food deprivation (Pierce *et al.* 2001). In a study on the stress response of fasted adult Atlantic salmon, it was found that plasma cortisol levels, a primary stress response marker, were moderately increased after 1 week of fasting but returned to initial values after 2 weeks of fasting (Waagbø *et al.* 2017). This could explain our significant findings after 1 week, but not 2 weeks. Juvenile chinook and chum salmon have also shown to be resistant to food deprivation, where a 2-week fasting period did not induce mortality or reduce condition factor (Ban *et al.* 1996, Snyder, 1980). In rainbow trout, 1 week of fasting significantly decreased their O_2_ consumption and CO_2_ excretion, while increasing ammonia excretion, however, after 2 weeks of fasting levels were not significantly different to control levels (Lauff and Wood 1996). This may have been similar in our study and may have caused the significant effects seen after 1 week of fasting and CO_2_ exposure, but not after 2 weeks.

These stocks experience a natural high CO_2_ zone in the Discovery Islands and Johnstone Strait every year, and they have been using this migration route for years on evolutionary time scales. As the water is so deeply mixed, there is a high degree of inertia on the environment experienced by fish in the TMZs, and the changes that they may experience with climate change is likely mostly in the long-term. Conversely, in the Strait of Georgia to the south and Queen Charlotte Strait north of the TMZ, fish are exposed to fluctuations in ocean conditions on seasonal and inter-annual scales.

It has been shown that fish experiencing naturally elevated CO_2_ conditions may have adapted to tolerate future global levels of OA (Frommel *et al.*, 2013, Lonthair *et al.*, 2017), and pre-exposure of the adults to high CO_2_ can have positive effects on the offspring (Allan *et al.*, 2014, Parker *et al.*, 2012). Therefore, pink salmon populations that experience high CO_2_ levels in their yearly migration may be pre-adapted to high CO_2_ levels. On a shorter time scale, tolerance to acute stressors is often dependent on pre-exposure to those stressors, and prior rearing in high temperature and low oxygen has been found to protect against acute increases in temperature and hypoxia in salmon early life stages (Del Rio *et al.*, 2019). Studies using hatchery-spawned and reared salmon (Del Rio *et al.*, 2019, Ou *et al.* 2015, Williams *et al*. 2019) may lose this phenotypic plasticity and therefore exhibit greater effects to climate stressors in relation to our study with wild-caught fish (Zhang *et al.*, 2016).

Pink salmon are one of the few species of anadromous Pacific salmon whose populations are still returning to spawn in strong numbers, while populations of most other salmon species have shown drastic declines in the North East Pacific (Beamish *et al.*, 2012, Sobocinski *et al.*, 2018). Compared to other species, pink salmon have an exceptional aerobic scope and temperature tolerance (Clark *et al.*, 2011). It may be that they are more resilient to climate stressors than other species of Pacific salmon. Alternatively, their life history may make them more robust to OA. It is also possible that the individuals we captured at Quadra Island are the survivors that have made it through the costly phase of smoltification and therefore are the strongest individuals.

## Conclusion

Contrary to our hypothesis, that pink salmon would be vulnerable to suboptimal ocean conditions, due to their small size, high surface to volume ratio and previously documented vulnerability to OA, juvenile pink salmon were not greatly affected by high CO_2_ conditions in this study. Previous exposure to high CO_2_ in the Strait of Georgia may have primed them for the CO_2_ conditions in our experiment, or the fish may have been able to rapidly acclimate to the CO_2_ levels used in this study that are similar to those already occurring in the TMZ. This study indicates that wild juvenile pink salmon may be robust to current and future OA. Whether this trait is unique for pink salmon, or all wild salmon populations undertaking the migration through high CO_2_ water, remains to be investigated.

## Funding

This work was funded by the Tula Foundation and Mitacs grant F19-00303 and a Natural Sciences and Engineering Research Council (NSERC) Discovery grant to C.J.B. (2018-04172).
